# Opportunities to address gaps in early detection and improve outcomes of liver cancer

**DOI:** 10.1093/jncics/pkad034

**Published:** 2023-05-05

**Authors:** Brian McMahon, Chari Cohen, Robert S Brown Jr, Hashem El-Serag, George N Ioannou, Anna S Lok, Lewis R Roberts, Amit G Singal, Timothy Block

**Affiliations:** Liver Disease and Hepatitis Program, Alaska Native Tribal Health Consortium, Anchorage, AK, USA; Hepatitis B Foundation, Doylestown, PA, USA; Department of Medicine, Division of Gastroenterology and Hepatology, Weill Cornell Medicine, New York, NY, USA; Department of Medicine, Baylor College of Medicine, Houston, TX, USA; Department of Medicine, Division of Gastroenterology, VA Puget Sound Health Care System, Seattle, WA, USA; Department of Internal Medicine, University of Michigan, Ann Arbor, MI, USA; Department of Internal Medicine, Division of Gastroenterology and Hepatology, Mayo Clinic, Rochester, MN, USA; Department of Internal Medicine, Division of Digestive and Liver Diseases, UT Southwestern, Dallas, TX, USA; Baruch S. Blumberg Institute and Hepatitis B Foundation, Doylestown, PA, USA

## Abstract

Death rates from primary liver cancer (hepatocellular carcinoma [HCC]) have continued to rise in the United States over the recent decades despite the availability of an increasing range of treatment modalities, including new systemic therapies. Prognosis is strongly associated with tumor stage at diagnosis; however, most cases of HCC are diagnosed beyond an early stage. This lack of early detection has contributed to low survival rates. Professional society guidelines recommend semiannual ultrasound-based HCC screening for at-risk populations, yet HCC surveillance continues to be underused in clinical practice. On April 28, 2022, the Hepatitis B Foundation convened a workshop to discuss the most pressing challenges and barriers to early HCC detection and the need to better leverage existing and emerging tools and technologies that could improve HCC screening and early detection. In this commentary, we summarize technical, patient-level, provider-level, and system-level challenges and opportunities to improve processes and outcomes across the HCC screening continuum. We highlight promising approaches to HCC risk stratification and screening, including new biomarkers, advanced imaging incorporating artificial intelligence, and algorithms for risk stratification. Workshop participants emphasized that action to improve early detection and reduce HCC mortality is urgently needed, noting concern that many of the challenges we face today are the same or similar to those faced a decade ago and that HCC mortality rates have not meaningfully improved. Increasing the uptake of HCC screening was identified as a short-term priority while developing and validating better screening tests and risk-appropriate surveillance strategies.

HCC is the third-leading cause of cancer death worldwide, resulting in an estimated 800 000 deaths annually (1). In the United States, from 1975 to 2012, hepatocellular carcinoma (HCC) was the only cancer to increase in both incidence and mortality in both men and women (2). In recent years, HCC mortality grew from 5.9 in 100 000 in 2010 to 6.5 in 100 000 in 2013, where it has remained (as of 2020 data) (3). If current trends continue, it is estimated that HCC will be the third-leading cause of cancer death in the United States by 2035 (4). The 5-year survival rate for HCC has not improved appreciably in the past 2 decades, hovering between 19% and 21% (it was 20.8% in 2020), whereas survival time has increased for most other cancers (3,5). The 2022 predicted 5-year survival rates ranks HCC as having the third-lowest survival rate among 27 cancer types (with only esophageal and pancreatic cancers having lower survival rates) (6).

Increased HCC surveillance use and improved effectiveness of screening tests for early HCC detection are essential; early detection is critical because prognosis is strongly associated with tumor stage at diagnosis. However, most HCC tumors continue to be diagnosed beyond an early stage when cure would be possible (7). In 2015, the Hepatitis B Foundation’s Princeton Workshop focused on identifying gaps and opportunities to improve the early detection of HCC (8). The Hepatitis B Foundation revisited this topic for the 2022 Princeton Workshop, convening 29 leading hepatitis B virus (HBV) and HCC experts to discuss the status of HCC in the United States. The workshop focused on the urgent need to improve the uptake and effectiveness of HCC surveillance in high-risk patients, discussing challenges and strategies toward improving early detection and mortality. Key takeaways from the workshop regarding challenges, opportunities, and action are summarized in Box 1.

## Screening and surveillance recommendations for HCC

Most HCC occurs in individuals with cirrhosis from underlying chronic liver disease, such as viral hepatitis (HBV or hepatitis C virus [HCV]), alcohol-associated liver disease, or nonalcoholic fatty liver disease (NAFLD). In patients with HBV or NAFLD, studies suggest that one-quarter to one-third of HCC can occur in the absence of cirrhosis (9-11). Although there has been progress in reducing the incidence of viral-related HCC, there has been an increase in the incidence of NAFLD-related HCC worldwide (12).

Current guidance from the American Association for the Study of Liver Diseases recommends surveillance for HCC in patients with Child-Pugh A or B cirrhosis and patients with Child C cirrhosis who are on a liver transplant waiting list. The recommended surveillance approach is semiannual ultrasound with serum alpha-fetoprotein (AFP) level (13). Similar guidance has been issued by the National Comprehensive Cancer Network (14) in the United States, the European Association for the Study of the Liver (15), and the Asian Pacific Association for the Study of the Liver (16). Some guidelines also recommend HCC surveillance for specific individuals without cirrhosis, such as those with HBV infection who are at higher risk of HCC due to sex, family history, age, and ethnicity or country of birth (17). HCC surveillance starts with identifying at-risk individuals to screen and performing guideline-concordant follow-up and treatment in those who screen positive.

The best data for HCC surveillance comes from a large randomized controlled trial from China among HBV-positive individuals demonstrating reduced HCC-related mortality (18). Although there have been no randomized controlled trials of HCC surveillance in patients with cirrhosis, systematic reviews have shown that surveillance for HCC is associated with improved detection of early-stage tumors, initiation of curative therapy, and increased overall survival (19-21).

## Suboptimal identification of at-risk population

Multiple factors affect the effectiveness of HCC screening and surveillance (22). The first step is identifying those at risk for HCC. Guidelines call for screening of patients with cirrhosis due to any cause and selected patients with chronic HBV infection; however, there are still many unanswered questions about which patients should be screened, what tools are most effective, and how often screening should occur. The current surveillance recommendations are “one size fits all.” However, there is a large gradation of HCC risk. For example, for people with chronic HBV, the risk of HCC varies with viral load, with a high viral load being a strong driver HCC risk (23), along with HBV genotype (24,25). Yet the approach to screening (tests used and frequency) is the same regardless of risk level. Some patients at risk are likely being overscreened while others are underscreened. There are also populations at risk not included in current guidelines because there are no studies that provide evidence-based data to allow a recommendation. Risk is dynamic, and there are questions about how often to reassess risk and if or when risk might be diminished enough to stop surveillance, for example, in patients with HBV or HCV with viral suppression or cure or who have cirrhosis with regression of fibrosis over time with treatment.

One of the most common barriers to implementing HCC surveillance is that screening for viral hepatitis and chronic liver disease is underperformed (26-28). It is estimated that 30% of the US population has NAFLD and 20% of those have nonalcoholic steatohepatitis (NASH), which puts them at risk of developing liver fibrosis (12,29). Thus, persons with risk factors for NAFLD (ie, type 2 diabetes; elevated BMI, especially if >30; signs of metabolic syndrome) should be screened for presence of advanced fibrosis or cirrhosis. In addition, the Centers for Disease Control and Prevention (CDC) and US Preventive Services Task Force (USPSTF) have now recommended universal 1-time testing for HCV for all US adults (30,31), and the CDC is proposing universal 1-time testing for HBV (32). Finally, all patients should be screened for alcohol usage with a rapid questionnaire such as the National Institute on Alcohol Abuse and Alcoholism (NIAAA) 1-question screen or 3-question Audit C (33,34). Those with evidence of heavy alcohol usage should be screened for evidence of liver disease. Once a person is found to have liver disease, assessment of fibrosis and HCC risk stratification should be performed.

## Improving HCC risk assessment and stratification

Risk-based surveillance (or “precision screening”), whereby an individual patient’s HCC risk is estimated and different screening strategies are recommended according to the level of risk, has been proposed to better target surveillance programs. Multiple HCC risk estimation models have been developed specifically for patients with HBV, such as PAGE-B (platelets, age, gender, and HBV), mPAGE-B (modified platelets, age, sex, and HBV), REACH-B (risk estimate for hepatocellular carcinoma in chronic hepatitis B), CU-HCC (Chinese University-HCC), and recently a machine learning model (Prediction of Liver cancer using Artificial intelligence-driven model for Network – hepatitis B, PLAN-B) (35). Risk estimation tools have been proposed for patients with cirrhosis or advanced chronic liver disease such as the Toronto HCC risk index (36), the aMAP (age-male-albumin-bilirubin-platelets) score (37), and risk models from the Veterans Affairs health-care system (38,39). There has also been increasing work in novel risk stratification via biomarkers or genetic risk scores, which appears promising but requires validation (40).

Risk stratification works best in those at risk of HCC and has a low positive predictive value when applied to the general population or other low-risk populations. Risk stratification also identifies people who may not benefit from surveillance because of having a very low risk of HCC. In the absence of risk-based surveillance strategies, providers often refer low-risk patients for screening for fear of missing the early stages of cancer (35).

There is an extensive list of risk factors for HCC that could be considered for use in risk calculators (41). Not all factors confer the same level of risk, and modeling is needed to obtain a multivariable score when incorporating multiple risk factors in calculators. Additionally, many risk calculators are meant to be used for specific risk groups, notably patients with chronic HBV infection, and may not be applicable to patients with liver disease due to other etiologies. Although universal risk calculators are desired, models incorporating specific characteristics of individual liver disease, such as HBV DNA levels in untreated with patients chronic HBV, may perform better than generic models. Results from risk calculators need to be linked to surveillance and prevention actions specific to the patient’s risk level. Finally, risk calculators have often been tested and internally validated in the same population; however, external validation is critical before widespread adoption.

Improved HCC risk calculators and algorithms are needed for integration into clinical care to aid providers in determining a patient’s level of risk for HCC and recommending risk-appropriate screening. In addition, the development of risk calculators that could be made available to patients should be investigated as an approach to raise awareness, motivate patients to seek care, and help them better understand their own risk for developing HCC.

## Suboptimal performance of current HCC screening tests

The effectiveness of HCC surveillance depends on whether the screening tests used can detect HCC at an early, treatable stage. The recommended screening tests (ultrasound and AFP) are widely available and low cost, but the sensitivity and specificity of these tests are suboptimal. In a noncirrhotic liver, the sensitivity of ultrasound for detecting small HCC lesions is estimated to be only 60% (42). In patients with cirrhosis, it is difficult to differentiate between nodules that might be benign vs HCC. Fat attenuates the ultrasound beam, and the performance of ultrasound for HCC surveillance has declined as obesity in the United States has increased (43,44). Further, the quality of an ultrasound is highly operator dependent (45). Studies suggest that when used alone, ultrasound can miss more than one-half of early-stage tumors (46). Using ultrasound in combination with AFP improves the sensitivity for early-stage HCC to 63%; however, this strategy still misses over one-third of early-stage HCC, highlighting a need for improved surveillance tests (46). The benefits of HCC surveillance must also be weighed against potential physical, financial, and psychological harms of false-positive results, although current data suggest these harms are mild in severity (47,48).

Although serum AFP level is commonly used for surveillance in those at risk, it has lower sensitivity for early detection of HCC and is more commonly elevated in advanced disease. Further, false positives occur (in association with liver inflammation and liver regeneration). Finally, some tumors do not produce AFP (42). Although AFP has historically been plagued by low specificity in the setting of active viral hepatitis, most recent data suggest this is less of a problem in nonviral etiologies of cirrhosis (49,50). AFP has been shown to be comparable with other biomarkers, for example, des-γ-carboxy-pro-thrombin (DCP) in HCC diagnosis, and may improve early detection when used in combination. AFP is often elevated in infiltrative HCC when ultrasound may be negative (51).

## Emerging HCC screening modalities

As efforts are being made to increase the use and uptake of current screening tools, there is a need for simultaneous development of better screening tests. Next-generation HCC screening will likely include improved biomarkers and/or advanced imaging techniques, such as abbreviated MRI (Magnetic Resonance Imaging) (aMRI) or radiomics.

### Biomarkers

In addition to AFP, a range of other protein-based serum biomarkers for HCC are currently in clinical use or under investigation; however, sensitivity is suboptimal, and no single biomarker is sufficient for early detection. Serum biomarkers are being tested in panels in combination with diagnostic algorithms to enhance detection of HCC. One example of a statistical model for HCC risk assessment in patients with chronic liver disease is GALAD, a serum biomarker-based model that incorporates sex, age, AFP, lectin-bound AFP (AFP-L3), and DCP (52,53). A recent study suggests that GALAD is more sensitive and specific than AFP in detecting preclinical HCC, and use of GALAD may prevent unnecessary MRI or CT (computerized tomography) tests in up to 54% of patients with cirrhosis (54). Another example is the Doylestown Plus algorithm, which builds on GALAD, incorporating sex, age, AFP, alkaline phosphatase, alanine aminotransferase, and a key substitution of fucosylated low–molecular-weight kininogen for the AFP-L3 in GALAD (55,56). Although fucosylated kininogen and AFP-L3 have similar sugar structures, AFP is present only in AFP-positive tumors, whereas kininogen is present in many more tumors. Spatial glycoproteomic studies of N-linked glycans are underway to identify sugar structures found in HCC tumors for potential use in developing a diagnostic platform (57). There have also been case-control studies suggesting that methylated DNA marker panels, used alone or in combination with protein biomarkers, can achieve high sensitivity for early-stage HCC detection (58,59). Several emerging biomarkers are also being studied for risk prediction, such as the Khan risk prediction assay, which uses a combination of proteins and free fatty acids for HCC risk assessment in patients with cirrhosis (60). It is important to note that all the emerging biomarker-based strategies still require further validation according to the Early Detection Resarch Network (EDRN) paradigm of biomarker validation before use in practice (61).

Other potential HCC biomarkers currently being studied are circulating RNA, including long noncoding RNA and micro RNAs, circulating tumor cells; cell-free DNA (cfDNA), and extracellular vesicle-based biomarkers. One can, for example, quantify total circulating cfDNA, assess fragmentation, look for somatic mutations in circulating cfDNA that are specific to HCC, or analyze the methylation of the circulating tumor DNA (ctDNA) fraction of cfDNA (62,63). ctDNA has been studied as a diagnostic and prognostic biomarker for HCC (64). Methylated DNA and chromatin fragments are shed from very early cancers, and ctDNA methylation in particular holds promise as an HCC early screening methodology. Panels of blood-based methylated cfDNA biomarkers associated with HCC have been shown to have high sensitivity for early-stage HCC. There is also emerging evidence for use of extracellular vesicle-based biomarkers in HCC surveillance (65,66). Products in development for HCC surveillance include multi-analyte tests with diagnostic algorithms that analyze cfDNA methylation in plasma, AFP, AFP-L3, and DCP levels in serum and patient age and sex to provide a qualitative screening result (58,59,67,68).

Multi-cancer early detection (MCED) tests that leverage cfDNA mutation-based and ctDNA methylation-based biomarkers have also shown high sensitivity for HCC in studies, both alone (69) and in combination with cancer-associated serum protein levels (70). MCED may prove to be especially important for diagnosis of HCC in persons who have a relatively low risk of HCC that does not justify routine surveillance on the basis of cost-effectiveness, such as persons with NAFLD without cirrhosis. The use of MCED panels in early detection of higher incidence cancers may substantially enhance early detection for less common cancers. Circulating tumor cells, although not sufficiently sensitive for early detection of HCC, could potentially be used for ongoing surveillance of patients after treatment to detect tumor recurrence after resection or tumor progression and metastasis after noncurative treatment.

The technology for developing optimized, robust HCC biomarker panels exists, and prospective studies are now needed to demonstrate reproducibility in a screening population compared with standard of care. Development and maintenance of longitudinal, high-quality, well-pedigreed biorepositories of HCC samples is also needed to facilitate clinical validation of new HCC biomarkers as they are identified (through prospective specimen collection and retrospective blinded evaluation, as in the Prospective Randomized Open, Blinded End-point, PRoBE study) (71).

### Abbreviated MRI

MRI is statistically significantly more sensitive than ultrasound in detecting HCC lesions between 1 and 3 cm (72). However, complete, multiphasic MRI is time consuming (30-45 minutes scanner time) and consequently costly, which reduces its cost-effectiveness as a screening modality in patients with cirrhosis. For this reason, aMRI protocols have been developed and are under assessment to determine their sensitivity and specificity for early detection of HCC (73-76). aMRI is a shortened MRI protocol that uses contrast but requires only 10 to 15 minutes. The primary aim of aMRI is to reduce scanner time, thus reducing costs, with minimal or no reduction in the sensitivity and specificity for detecting early-stage HCC compared with a complete MRI. To achieve this, aMRI protocols include only limited sequences, specifically the sequences that are important for HCC detection (eg, only T1-weighted precontrast and dynamic contrast-enhanced images obtained after contrast injection). Early data suggest that aMRI retains high sensitivity and specificity for early-stage HCC, particularly in patients with NAFLD-related liver disease, although these findings still require validation in large cohort studies (77,78). Other issues of patient acceptance, radiologic capacity, and reimbursement (aMRI is currently not accessible for routine care, and there is no billing code for insurance reimbursement) still need to be resolved.

### Radiomics, deep learning, and neural networks

Another emerging technology with the potential to increase early detection of HCC is radiomics. Although radiologists review many images per patient, only a small fraction of the imaging features are used, and visual interpretation is subject to radiologists’ experience. Radiomics leverages mathematical models and artificial intelligence to extract a large number of imaging features. These imaging data are then synthesized with clinical data to aid clinicians in HCC diagnosis and prognosis (79-82).

Deep learning tools, such as neural networks, are also being studied for a potential role in detecting HCC in clinical images. Neural networks use algorithms to help computers make decisions, learning through execution. In early studies, neural networks demonstrated high levels of sensitivity and specificity in classifying liver tumors (comparable with radiologists) requiring only milliseconds per lesion (83-86).

Radiomics and neural networks would likely be used to assist radiologists by highlighting concerning images for a more careful review and are not meant to replace radiologists. Radiomics has been criticized as a “black box” method, and external validation can be difficult (87). Global standards that could be applied across analyses are needed.

## Underuse of HCC surveillance

Despite the demonstrated benefit of HCC surveillance, use is low (88,89). A recently published systematic review showed that among people with cirrhosis in the United States, an estimated 24% overall received HCC surveillance (88). Multiple barriers persist (90) at both the provider and patient levels (91,92). For those who have been identified as being at risk for HCC, screening can be inconvenient, and lack of insurance coverage and/or considerable out-of-pocket costs are a concern (93). The recommended frequency of screening can lead to “surveillance fatigue”; ultrasound requires fasting; and patients report additional challenges, including low awareness about the need for HCC surveillance, transportation challenges, difficulty scheduling, and uncertainty about where to obtain an ultrasound (94,95). Some patients are resistant to screening because they are uncertain of its benefit, face sociocultural barriers to screening and health-care access, or are fearful of being diagnosed with cancer (96-100). A recent survey found that although patients with cirrhosis were often very concerned about developing HCC, many believed that ongoing surveillance was not needed if they felt well and their physical examination and laboratory results were normal (94). Poor health literacy and medical mistrust can also contribute to failures across the HCC care continuum, including screening (100).

At the provider level, key barriers to effective HCC surveillance include providers not diagnosing HBV infection or other underlying chronic liver disease before the presentation of complications (eg, decompensated cirrhosis), not being aware of the benefits of surveillance, not understanding which patients need it, and not ordering screening tests for those with known cirrhosis (101,102). The screening process also breaks down when there is inadequate follow-up of detected lesions or failure to provide appropriate definitive diagnosis and treatment (103).

Primary care providers should play a role in conducting HCC surveillance because there are not enough specialists to take on this task and only a small proportion of patients with cirrhosis are under the care of a hepatologist. However, primary care providers report low awareness of surveillance guidelines and experience logistical barriers such as competing clinical concerns (101). In recent surveys, most primary care providers said they saw patients with cirrhosis, but less than one-half recommended these patients be screened for HCC (104).

Many people in the United States remain uninsured or underinsured, which affects their access to routine care (where underlying liver disease could be diagnosed) and their uptake of HCC screening. For individuals with health insurance, twice-yearly ultrasound screening for HCC is not specifically recommended by the USPSTF, which affects the extent of coverage by insurers. The lack of integrated electronic health record (EHR) systems can hamper the ability of providers to obtain reports of patients’ ultrasound exams and other testing if they are conducted at facilities outside the provider’s health system. System-wide stress on the health-care infrastructure can also result in overall reduced rates of screening for diseases, as was observed during the height of the COVID-19 pandemic (105).

## Increasing the uptake of HCC screening

The need to increase the use and uptake of HCC screening was identified as a priority by workshop participants. There was much discussion of the need to raise awareness of patients, providers, and the public about liver cancer in general, who is at risk for HCC, and the critical importance of HCC surveillance for those at increased risk. Messaging needs to convey that there is hope because many people still think liver cancer is always fatal and do not know that HCC can often be prevented or that curative treatments for HCC are readily available when diagnosed early. Because HCC is asymptomatic when tumors are small, patients and providers need to understand that early diagnosis of HCC increases curative treatment options and overall survival.

Patient-directed education should discuss the need to be aware of HCC risk as well as the importance of scheduling and completing testing and attending follow-up appointments. The importance of discussing screening results with their provider and that repeating testing at regular intervals (6 months) is crucial in addition to undergoing further assessment if an abnormality is found should be emphasized to patients. Provider-directed education should address identification of patients with potential of underlying liver disease (screening for HBV and HCV, alcohol use, and NAFLD) and among those with underlying liver disease who have early cirrhosis and are at risk for HCC. Once a provider has diagnosed cirrhosis, they need to be informed on applying risk stratification strategies for those who need to be screened for HCC, which HCC screening tests to order, and the importance of repeating the screening cycle at recommended intervals. Increased awareness is also needed regarding updates to the CDC recommendations for HBV and HCV screening, respectively (32).

From a systems perspective, strategies discussed for improving uptake of HCC screening included EHR-embedded clinical decision support tools to identify at-risk patients and provide order sets for HCC screening (106) as well as automated reminders to patients and providers when screening is recommended or due. For example, a 30-year program at the Alaska Native Health Care System sends reminder letters every 6 months to persons with underlying risk factors to have blood work and liver ultrasound. They also send reminder letters to providers (21). Outreach and care navigation is an important tool to help overcome patient barriers to accessing screening (107,108). It is crucial to ensure that screening test results are readily accessible (eg, via improved data sharing or integration of EHR systems) to both providers and patients to keep patients engaged. Implementing quality metrics for HCC screening and care to monitor the outcome of surveillance programs is also important. Although it would be unethical to conduct a randomized controlled trial that compares periodic screening with no screening, randomized controlled trials (such as the Preventing Liver Cancer Mortality through Imaging with Ultrasound vs. MRI, PREMIUM study) (109) to compare different screening modalities would be beneficial. Such studies could collect the robust evidence needed to support a USPSTF recommendation for HCC surveillance as well as identify more cost-effective screening strategies. It was also noted that universal health coverage would better enable access to and implementation of HCC surveillance.

## Moving forward

Princeton Workshop participants were disappointed in the lack of progress made since the 2015 meeting and the overall lack of urgency dedicated to addressing the continued increase in incidence and poor survival of HCC. Effective HCC surveillance is essential for saving lives, but screening to identify persons at risk for HCC and enrollment of these persons in a systematic program of ongoing surveillance for early detection of HCC continues to be underused. This is due to a range of challenges and barriers, which have been discussed for decades. Participants agreed that understanding HCC risk and risk assessment has matured and that HCC surveillance is now at an inflection point. New technologies are being studied that could transform early detection, diagnosis, and prognosis. This is an age of opportunity to improve detection and outcomes for patients with HCC, and there is much to do. Participants call for increased investment in early detection and surveillance research while implementing policies and practices that can help improve early detection of HCC using the tools we have now. We must act swiftly to prioritize early detection of HCC to save lives.

Box 1. Workshop highlights^a^

**Key takeaways**

- Little progress has been made in the past 7 years toward improving surveillance and early detection of HCC in the United States. Neither HCC surveillance nor mortality rates have meaningfully improved, and it is critical that we act swiftly to prioritize early detection of HCC to save lives.
- While there is a need for future research, the emphasis must be put on implementing the data and screening tools available now. We cannot wait for randomized controlled trials comparing different screening strategies, which take years, and randomly assigning at-risk persons to surveillance vs nonsurveillance is unethical.
- Systematic reviews have shown that surveillance for HCC is associated with improved detection of early-stage tumors, receipt of curative therapy, and overall survival. There is an urgent need to identify those at risk for HCC, improve and systematize HCC surveillance, and implement effective treatment for those detected with early-stage HCC.
- Abbreviated MRI has appeal as a novel screening strategy for HCC but needs to be investigated in randomized trials compared with liver ultrasound. New noninvasive biomarkers geared toward early detection of HCC and risk stratification are needed and important.

**Key challenges for HCC screening and surveillance**

- Many patients diagnosed with underlying liver disease are not in regular, longitudinal care and are therefore not being screened for HCC.
- Rates of HCC screening remain low, even among those identified as at risk.
- Sensitivity and specificity of the recommended screening tests (ultrasound with or without AFP) are suboptimal.

**Improving HCC surveillance**

- Patients with liver disease should seek regular monitoring, understand their risk of HCC, discuss surveillance with their provider, and adhere to surveillance appointments as recommended.
- Providers should identify patients at risk for HCC, recommend surveillance as appropriate, review results, and follow up abnormal results as needed.
- Health systems should implement risk assessment algorithms to identify patients at risk for HCC (eg, embedded as clinical decision support in EHR systems), send automated screening reminders to patients and providers, provide care navigation, and implement a quality metric for HCC screening.
- Researchers, clinicians, patients, and advocates need to work together to generate a national sense of urgency that will lead to change in policy and practice to improve HCC surveillance.

**Areas for further study and action**

- Conduct more studies to compare different screening modalities, including new biomarkers, MRI, and aMRI. Standardizing terminology and criteria will be a necessary step.
- Develop more data to support a USPSTF recommendation for HCC surveillance.
- Bring primary care providers to the table as allies in HCC screening.
- Develop an HCC risk calculator for use by and available to patients.
- Investigate approaches that address patient and provider challenges to improve implementation and uptake of HCC surveillance.
- Leverage knowledge from other cancers about effective screening tools and risk stratification.
- Develop and maintain longitudinal, high-quality, well-phenotyped biorepositories of HCC samples and prospectively collected sera and cells from patients at risk for HCC to:
  - Facilitate clinical validation of potential new HCC biomarkers as they are identified
  - Evaluate new markers retrospectively using established biobanks to ascertain when they first appear, before HCC diagnosis to evaluate their usefulness as a tool for early detection
  - Test new markers prospectively in at-risk persons to evaluate their effectiveness to detect HCC early at potentially curable stage

^a^AFP = alpha-fetoprotein; EHR = electronic health record; HBV = hepatitis B virus; HCC = hepatocellular carcinoma; HCV = hepatitis C virus; NAFLD = nonalcoholic fatty liver disease; USPSTF = US Preventative Services Task Force.

## Data Availability

No new data were used or generated for the writing of this commentary. The agenda for the 2023 Princeton Workshop will be made available upon request.

## Notes

for the 2022 Princeton Workshop Participants (see Acknowledgements)
